# Frequency of Black and American Indian/Alaskan Native US Residents Screened for Firearm Access

**DOI:** 10.1007/s40615-025-02334-8

**Published:** 2025-03-10

**Authors:** Allison E. Bond, Taylor R. Rodriguez, Gretchen Goldman, Jayna Moceri-Brooks, Daniel C. Semenza, Michael D. Anestis

**Affiliations:** 1 The New Jersey Gun Violence Research Center, Rutgers University, 683 Hoes Ln, Piscataway, NJ 08854 USA; 2 Department of Psychology, Rutgers University, 683 Hoes Ln, Piscataway, NJ 08854 USA; 3 School of Nursing, Duquesne University, Pittsburgh, PA USA; 4 Rory Meyers College of Nursing, New York University, New York, NY USA; 5 Department of Urban-Global Public Health, Rutgers University, 683 Hoes Ln, Piscataway, NJ 08854 USA; 6 Department of Sociology, Anthropology, and Criminal Justice, Rutgers–Camden, Camden, USA

**Keywords:** Firearms, Suicide prevention, Healthcare providers, Screening for access, Firearm safety

## Abstract

**Objective:**

This study evaluates Black and AIAN individuals’ self-reported history of being screened for firearm access by healthcare providers, and identifies factors that influence screening.

**Methods:**

A cross-sectional, nationally representative survey of included 3015 Black and 527 AIAN adults in the US. Participants were recruited via probability-based sampling.

**Results:**

Among Black participants, 13.1% and among AIAN participants, 18.4% reported being screened for firearm access. Of the participants who reported being screened, most have been by mental healthcare providers or primary care physicians. Factors associated with higher screening odds in Black adults included history of suicidal ideation or mental health treatment, current firearm access, younger age, and having children at home. For AIAN participants, a lifetime history of mental health treatment or identifying as female increased screening odds.

**Conclusion:**

Black adults report infrequently being screened for firearm access by healthcare providers. Identifying screening barriers and fostering discussions on firearm safety in healthcare settings are important next steps for firearm injury prevention efforts.

## Introduction

Suicides account for 60% of all firearm deaths within the United States (US), and firearms are used in over 50% of suicide deaths [1]. Firearms are the most lethal method for suicide, with an 85–95% fatality rate [1]. This means that when someone attempts suicide with a firearm, they rarely survive. Research on method substitution in suicide (e.g., utilizing a method other than the one planned due to inability) is ambiguous [2]. Given this, research has sought to determine ways to reduce firearm suicides within the US. One approach for accomplishing this goal is the promotion of secure firearm storage—storing a firearm unloaded, locked, and separate from ammunition—associated with reduced risk for suicide [2]. Although there is an association between secure storage and suicide risk, most firearm owners do not engage in secure storage [3]. Engaging in secure firearm storage before a suicidal crisis emerges is essential, given that those who attempt suicide think about suicide for as little as 10 min before they act [4]. One way to promote secure firearm storage is through lethal means counseling. Lethal means counseling refers to practices that include identifying risk factors for suicide, speaking with patients to identify access to lethal means (e.g., firearms, medications), safety planning for suicide, discussing firearm safety, secure firearm storage, ways to limit access to firearms and other methods, and providing locking devices [5]. In suicide prevention broadly, lethal means counseling shows promise for reducing risk and is often embraced by clinicians. In firearm suicide prevention specifically, lethal means counseling has been found to increase secure firearm storage habits [6]. An understanding of who owns firearms, their attitudes and behaviors to secure firearm storage, and barriers towards firearm screening and lethal means counseling is needed to effectively administer lethal means counseling interventions.

Healthcare providers are well conduct lethal means counseling, specifically engaging in conversations on secure firearm storage with those at risk for suicide. Previous research has demonstrated that 83% of those who die by suicide interact with healthcare in the year prior to their death [7]. The National Strategy for Suicide Prevention specifically states that limiting access to lethal means as a key priority for reducing suicide risk. Additionally, professional organizations recognize the role providers have in screening for firearm access; the American Academy of Pediatrics has put forth recommendations for pediatricians to conduct routine firearm screening with parents during well-child visits [8]. Although these recommendations are in place, many providers do not screen for firearm access. The lack of screening may be due to a variety of reasons including lack of guidelines [9], lack of time [10], and belief that patients would not be receptive [11].

Previous research indicates that, even among groups at high risk for suicide, screening for firearm access rarely occurs. Specifically, only 9.2% of Veterans using Veterans Health Administration services report being screened for firearm access by a clinician [12] and only 18% of adults who present to the emergency department (ED) for suicide risk have been screened for firearm access [13]. A study conducted among a nationally representative sample found that only 7.5% of adults who live firearm owning households have discussed firearm safety with a healthcare provider [14]. A recent study expanded upon previous work and found that among a representative sample from five states 17.1% report having been screened for firearm access [15]. Additionally, several factors were associated with increased odds of being screened. Specifically, lifetime suicidal ideation, firearm ownership, mental health treatment, having children under the age of 18 in the home, being male, and identifying as White were associated with significantly increased odds of being screened for firearm access. Concerningly, these findings suggest that racial and ethnic minorities may be less likely to be screened for firearm access by healthcare providers, and in turn less likely to receive resources for secure firearm storage.

The potential decreased rates of screening among racial and ethnic minorities are especially concerning when considering that Black and American Indian/Alaskan Native (AIAN) individuals are disproportionately impacted by firearm injury and death. Specifically, Black men have the highest age adjusted rate of firearm violence (51.98 deaths per 100,000; compared to 4.75 per 100,000 among White men) [16]. Concerningly, the heightened risk of death among Black individuals is consistent across healthcare broadly and is not unique to firearm violence. Black women are three times more likely to die from pregnancy related complications than white women [17]. A similar picture unfolds when examining AIAN healthcare. AI individuals are three times more likely to be diagnosed with type 2 diabetes compared to white individuals [18]. Additionally, racial and ethnic minorities report more barriers to healthcare than their white counterparts [19]. These statistics highlight that health disparities are pervasive in the US, and firearm suicide risk may be another area in which Black and AIAN individuals are not receiving the same level of care as white individuals.

Additionally, the firearm suicide rate among Black American residents has risen; the firearm suicide rate among Black youth increased 115% from 2011 to 2020, and rose an additional 1.36% in 2021 [1]. In addition, the firearm purchasing surge that began in 2020 saw an unprecedented number of Black individuals purchasing firearms than previous years [20]. The increase in firearm availability is especially concerning given that it increases the risk for suicide. Similarly, the AIAN community experiences high rates of firearm fatalities, especially suicide. AIAN individuals (10.6 per 100,000) have comparable rates of firearm suicide to white individuals (11.1 per 100,000), and higher rates than all other racial groups [21]. Between 2019 and 2020, the firearm suicide rate increased by 42% among AIAN individuals, which represented the largest increase for any racial subgroup [16]. The high rates of firearm ownership, and injury and death among Black and AIAN individuals are concerning and speak to the need for lethal means counseling within these communities. However, while research has examined the frequency of screening for firearm access among nationally representative samples—where Black and AIAN individuals are the minority—research has yet to examine the frequency with which screening for firearms occurs within a large, representative sample of Black and AIAN communities and factors that increase the likelihood of being screened for firearm access.

The present study seeks to fill this gap by determining the frequency with which the Black and AIAN communities report being screened for firearm access by healthcare providers within our sample population. Additionally, we examine factors associated with odds of being screened. Lastly, this study examines the types of providers (e.g., mental health, primary care) that the Black and AIAN community report most often screened for firearm access. Findings can help clarify the frequency with which screening for firearm access occurs. Ultimately, this can promote secure firearm storage conversations within healthcare settings, which may increase secure firearm storage and decrease suicide rates among those who identify as Black and AIAN. While the present study is not specifically examining race-based disparities, findings provide initial insight into potential disparities that may exist and support future investigation.

## Methods

Nationally representative data were collected from KnowledgePanel (KP) via Ipsos between April 12 and May 4, 2023 (completion rate 59%). The samples included Black (*n* = 3015*)* and AIAN (*n* = 527) adults in the US. Participants could select more than one racial identity and 15 participants overlap between the two samples. The Institutional Review Board at Rutgers University approved the procedures and all participants provided informed consent.

Data weights were calculated by Ipsos first by reflecting selection probabilities, then by weighting to geodemographic distributions based on the 2022 Census Population Survey for each sample. All analyses were conducted in the separate samples and appropriate weights were used for each.

Demographics were obtained from items embedded within the KP profile, including age, sex, and presence of children under the age of 18 in the home. Firearm access was assessed with a single item, “Is there typically a firearm stored in or around your home?” Participants were asked, “Has a healthcare provider (physical or mental health) ever asked you if you have access to firearms?” Those who confirmed being screened for firearm access were further asked to specify the types of provider[s] who screened them. Options included primary care physicians, pediatricians, children’s mental healthcare providers, primary care nurses, ED physicians, ED nurses, other providers, and school nurses. Lifetime mental health treatment utilization for any reason (e.g., therapy, medication, assessment, other), current access to a firearm (i.e., firearm in or around the home), and marital status were all self-reported via single items. The Self-Injurious Thoughts and Behaviors Interview-Revised (SITBI-R) [22] was utilized to assess history of suicidal thoughts in participants’ lifetime.

### Data Analytic Plan

Descriptive statistics in each sample assess the overall frequency at which participants report being screened for firearm access, including among specific provider types. Logistic regressions within each sample were utilized to determine factors associated with being asked about firearm access by any healthcare provider.

## Results

### Black Sample

Table 1 describes the study sample. Among those who identify as Black, 13.1% reported being screened for firearm access by a healthcare provider. As can be seen in Table 4, screening most often occurred by the participants’ mental healthcare providers (50.1%) and primary care physicians (48.1%), followed by pediatricians (12.2%), children’s mental healthcare providers (9.3%), primary care nurses (8.0%), ED physicians (7.3%), ED nurses (5.7%), other providers (4.8%), and school nurses (1.2%).

**Table 1:** Weighted sample characteristics

	Black	American Indian/Alaskan Native
	*N* = 3015	*N* = 527
Age		
*M*(*SD*)	46.34 (16.25)	46.20 (16.08)
Range	18–94	18–97
Sex		
Male	1369 (45.4%)	247 (46.9%)
Female	1646 (54.6%)	280 (53.1%)
Marital status		
Not currently married	1936 (64.2%)	271 (51.4%)
Married	1079 (35.8%)	256 (48.6%)
Children in home		
No	1982 (65.7%)	349 (66.1%)
Yes	1033 (34.3%)	178 (33.9%)
Mental health treatment		
No	1966 (65.6%)	308 (58.9%)
Yes	1031 (34.4%)	215 (41.1%)
Suicidal ideation		
No	2306 (77.0%)	340 (64.6%)
Yes	689 (23.0%)	186 (35.4%)
Firearm access		
No	2080 (69.0%)	286 (54.2%)
Yes	909 (30.2%)	238 (45.2%)
Refused	26 (0.9%)	3 (0.5%)

As shown in Table 2, a lifetime history of suicidal ideation (OR = 2.249 [1.756, 2.879]), a lifetime history of mental health treatment (OR = 3.378 [2.645, 4.314]), being younger in age (OR = 0.990 [0.981, 0.998]), having access to a firearm (OR = 1.398 [1.095, 1.785]), and having children 17 years old and younger in the home (OR = 1.395 [1.094, 1.778]) were associated with increased odds of being screened for firearm access by healthcare providers. As shown in Table 3, 26.9% of those with suicidal ideation, 25.4% of those with a history of mental health treatment, 16.9% of those with children in the home, and 15.2% of those who have access to a firearm reported being screened for firearm access. There were no significant differences in terms of sex or marital status.

**Table 2:** Logistic regression examining variables associated with healthcare providers asking about access to firearms

	Black/African American			American Indian/Alaskan Native		
	*p*	OR	CI	*p*	OR	CI
Mental health treatment	< 0.001	3.378	2.645, 4.314	< 0.001	4.134	2.462, 6.942
Suicidal ideation	< 0.001	2.249	1.756, 2.879	0.714	1.104	0.651, 1.872
Marital status	0.145	1.204	0.938, 1.546	0.735	1.090	0.662, 1.795
Sex	0.078	1.234	0.976, 1.560	0.049	1.631	1.002, 2.657
Age	0.013	0.990	0.981, 0.998	0.324	1.009	0.991, 1.027
Kids in home	0.007	1.395	1.094, 1.778	0.212	1.414	0.821, 2.436
Firearm access	0.007	1.398	1.095, 1.785	0.106	1.494	0.918, 2.434

*Mental health treatment, suicidal ideation, marital status, kids in the home, and firearm owner are coded as 0 = no, 1 = yes. Sex is coded as 0 = man, 1 = woman. Age is coded on a continuum from 18 to 97 years old

**Table 3:** Percentages examining who was screened for firearm access among a subsample of those who endorsed the listed variables

	Black/African-American		American Indian/Alaskan Native	
	%	CI	%	CI
Mental health treatment	25.4%	0.229, 0.282	30.0%	0.244, 0.366
Suicidal ideation	26.9%	0.237, 0.303	23.5%	0.180, 0.301
Married	13.7%	0.117, 0.158	19.2%	0.079, 0.157
Male	10.9%	0.093, 0.126	15.2%	0.113, 0.203
Female	15.1%	0.135, 0.169	21.2%	0.167, 0.262
Kids in home	16.9%	0.096, 0.136	21.0%	0.158, 0.278
Firearm access	15.2%	0.823, 0.870	19.9%	0.151, 0.252

*Indicates the percentage among those who responded “yes” to the variable in the column that were or were not screened for firearm access. Age was not examined given that it is a continuous variable

### American Indian/Alaskan Native Sample

Among those who identified as AIAN, 18.4% reported being screened for firearm access by a healthcare provider. As can be seen in Table 4, screening most often occurred by primary care physicians (57.6%) and participants’ mental healthcare providers (48.0%), followed by pediatricians (13.2%), children’s mental healthcare providers (13.2%), ED physicians (7.5%), primary care nurses (7.4%), ED nurses (6.6%), other providers (2.2%), and school nurses (0.5%).

**Table 4:** Types of healthcare providers who were reported to have screened for firearm access

	Black/African-American		American Indian/Alaskan Native	
	%	CI	%	CI
Mental healthcare	50.1%	0.453, 0.552	48.0%	0.377, 0.573
Primary care physician	48.1%	0.433, 0.532	57.6%	0.478, 0.672
ED physician	7.3%	0.051, 0.103	7.5%	0.033, 0.136
Pediatrician	12.2%	0.092, 0.157	13.2%	0.077, 0.212
Child’s mental healthcare	9.3%	0.068, 0.126	13.2%	0.077, 0.212
Primary care nurse	8.0%	0.055, 0.108	7.4%	0.033, 0.136
ED nurse	5.7%%	0.038, 0.085	6.6%	0.026, 0.123
School nurse	1.2%	0.005, 0.028	0.5%	0.001, 0.047
Other	4.8%	0.030, 0.073	2.2%	0.004, 0.064

*Sample limited to those who had reported being screened for firearm access

As depicted in Table 2, a lifetime history of mental health treatment (OR = 4.134 [2.462, 6.942]) and identifying as female (*OR* = 1.631 [1.002, 2.657]) were associated with increased odds of being screened for firearm access by healthcare providers. Table 3 indicates that 30.0% of those with a history of mental healthcare and 21.2% of women reported being screened. There were no differences in terms of marital status, age, children in the home, suicidal ideation, or firearm access.

## Discussion

This study sought to determine the proportion of those who identify as Black or AIAN and reported being screened for firearm access by healthcare providers and determine demographic factors that impact the odds of being screened. The study expanded upon Bond and colleagues by examining the types of providers that were most often reported as screening for firearm access and focusing on nationally representative samples of individuals who identify as Black or AIAN.^11^ The findings indicate that Black and AIAN individuals report being rarely screened for firearm access by healthcare providers (13.1%; 18.4%). AIAN individuals report being screened at similar rates as White individuals, while screening among Black individuals was reported at a lower rate. While research indicates a low rate of firearm access screening overall by healthcare providers [5, 15], findings from this study provide further indications that Black individuals are likely being screened at disproportionately lower rates. These findings highlight the racism, barriers to adequate care, and implicit bias that may likely be present within the healthcare system.

Institutional racism is embedded in the healthcare setting and often results in Black individuals feeling unsafe in medical settings and receiving less quality care than white individuals [23, 24]. The findings in the present study suggest that the racism embedded within the healthcare system impacts screening for firearm ownership. A specific form of the racism that may impact screening rates is implicit bias—or negative attitudes towards specific racial groups—among healthcare providers [25]. While the specific biases are unknown, it may be that healthcare providers are falsely assuming that Black individuals do not own firearms and therefore do not need to be screened for firearm access, or that Black individuals who do own firearms own them illegally and will not disclose ownership status. Many Black individuals own firearms [26] and, regardless of the legality of the firearm, can benefit from screening for firearm access and lethal means counseling. Providers’ implicit bias against Black Americans results in low rates of firearm screening, which in turn withholds evidence-based tools (e.g., lethal means counseling) that can help reduce firearm injury and death. In addition to implicit bias, other structural factors like lower rates of health insurance among racial minorities (Sohn, 2018) may impact rates of screening. Specifically, those who are uninsured or underinsured may attend fewer medical appointments, and therefore have less of an opportunity to be screened for firearm access. Another factor may be that there is less mental healthcare provider presence in Black communities, which in turn may lead to more stigma, less disclosure, and then fewer opportunities to be screened for firearm access [27]. To address some of these disparities and increase the rates of screening among Black and AIAN individuals, healthcare providers should complete evidence-based training on racial health disparities, racism in healthcare, and microaggression throughout their career. However, given that institutional racism may continue to be embedded in the healthcare system, universal screening for firearm access should be employed. Universal screening for firearm access would require that providers screen every patient—regardless of racial identity—for firearm access.

In this study, we found differences in firearm access screening practices among healthcare providers between the two samples. Black individuals report being more likely to be screened if they had a history of suicidal ideation and mental health treatment, were younger, had access to a firearm, and had children under the age of 18 in the home. These findings align with previous research examining patient characteristics that increase the likelihood of being screened for firearm access by a healthcare provider [14], indicating that similar factors are prompting providers to screen for firearm access among Black individuals as White individuals, although the screening is being reported to occur less frequently. Of note, roughly a quarter of those with suicidal ideation (26.9%) and those with a history of mental health treatment (25.4%) reported being screened for firearm access. Therefore, even though perceived risk of suicide may be promoting some providers to screen for firearm access, the vast majority of those experiencing ideation and seeking treatment are not being screened for access. This is especially concerning among the Black population given that rates of firearm ownership and suicide have increased substantially over the past few years [16, 20].

Individuals identifying as AIAN reported being more likely to be screened for firearm access if they had a history of mental health treatment and identified as female. This differs from previous research, which demonstrated that, among White individuals, males were more likely than females to be screened for firearm access [15]. However, it is important to note that only 30% of those with a lifetime history of mental health treatment and 21.2% of women reported being screened for firearm access. This finding indicates that the vast majority of those in mental health treatment and women are not screened for access. Marital status, age, number of children in the home, suicidal ideation, and firearm access were not associated with increased odds of being screened for firearm access within the AIAN sample. This finding may be explained by the fact that homicides and intimate partner violence are disproportionately high among AIAN women compared to White women [28], prompting healthcare providers to screen for firearms among women. Alternatively, when AIAN women disclose experiencing IPV during their visit, it may prompt some healthcare providers to screen for firearm access.

In both samples, a history of suicidal ideation and previous mental health treatment were associated with an increased likelihood of being screened for firearm access. These findings aligns with the common practice for risk assessments—such as the Columbia Suicide Severity Rating Scale [29]—to be administered in mental healthcare settings, and especially when a client endorses suicidality, and a component of risk assessments is assessing for lethal means, including firearms. However, even among Black and AIAN individuals with ideation and in treatment, screening rarely occurred. Given the low rates of screening for firearm access within these populations, there is a disparity in Black and AIAN individuals being provided the standard of care—which includes lethal means assessments, especially when they are presenting with concrete risks for suicide.

Among those who reported screening, they most often referenced mental health providers and primary care physicians as the type of providers who screened for firearm access. Among the AIAN sample, those who reported screening most often referenced primary care providers, and within the Black sample those who reported screening referenced primary care providers and mental healthcare providers at comparable rates. Among those who reported screening, pediatricians, children’s mental healthcare providers, primary care nurses, ED physicians, ED nurses, other providers, and school nurses were rarely referenced as screening for firearm access in both the Black and AIAN samples. Given that primary care providers and mental healthcare providers are the most referenced sources to screen for access, it is important to provide these professionals with additional support (e.g., training on lethal means counseling) to increase the frequency at which they are screening. In turn, this may lead to more individuals being screened for firearm access, which can increase firearm safety and reduce risk for injury and death. However, given that the majority of those who die by suicide with a firearm do not interact with mental healthcare [30], it is important to identify barriers that result in other healthcare providers not screening for access. Specifically, distributing resources (e.g., Lock2Live, safe firearm storage maps) to those tasked with screening for firearm access may help increase provider comfort regarding screening, and provide them with a tangible resource to give a patient who reports owning a firearm. Additionally, distributing secure storage devices is a potential avenue for increasing adherence with secure storage recommendations. Research has found that firearm owners are receptive to receiving secure storage devices from physicians [31], and that providing locking devices to firearm owners is associated with more secure storage habits [12]. Therefore, in addition to educating patients about secure storage and engaging in lethal means counseling, having healthcare providers disseminate secure storage devices may increase adherence with storage recommendations. To do this effectively, advocacy efforts are needed to ensure funding is available for both the training of providers and the procurement of secure storage devices to distribute.

### Public Health Implications

The low firearm screening rates among Black and AIAN individuals and high rates of firearm injury/fatalities within these populations highlight significant opportunities to increase screening efforts. The findings demonstrate that among those who are screened for firearm access, nurses are rarely referenced as the source who provided the screening, despite health promotion being a tenant of their profession [32] and being well positioned to establish rapport with patients and screen for firearm access. The field of nursing has recognized its important role in screening for firearm access and the American Academy of Nursing and the American Nurses Association released position statements in 2023 committing to preparing and strengthening nurses to address firearm violence through education and training on the topic. Providing nurses with training on lethal means counseling may bolster their comfort screening for access and may result in an increase in secure firearm storage among their patients.

In line with the American Academy of Pediatrics guidelines on firearm screening [33], pediatricians also have an opportunity to universally screen for firearms at every visit and provide lethal means counseling. In 2019, an American Academy of Pediatrics survey revealed that most pediatricians recognize firearm violence prevention as a priority, believe they should ask parents about firearm access, and feel that anticipatory guidance about firearm injury prevention can help reduce firearm injuries and deaths. However, many pediatricians cite lack of time, lack of proper training, and minimal resources as barriers preventing them from conducting firearm screening [33].

Additionally, many AIAN individuals receive care through the Indian Health Service (IHA). The IHA functions similarly to other major healthcare organizations, like VA and Kaiser Permanente. However, the VA and Kaiser Permanente have researchers focused on reducing rates of firearm injury and death among the communities they serve, which has resulted guidelines for screening for firearm access. To the best of our knowledge, the IHA has not put forth guidelines regarding screening for firearm access. Structural racism has resulted in members of the AIAN community being excluded from higher education and research, which leads to a lower percentage of AIAN individuals conducting research on firearm injury and death among their community. Therefore, addressing systemic barriers to education and participation in the research process may help to develop culturally informed firearm screening guidelines that would increase rates of screening among the AIAN community, and in turn reduce suicide rates.

Therefore, we recommend that curriculum on this topic become standardized within healthcare systems and community healthcare practices so that providers receive repetitive training with the same frequency and priority as other topics, such as infection control. For example, a study conducted by Sale et al. found that mental health providers who completed training on lethal means counseling had greater confidence in their ability to counsel patients and reported an increase in the belief that lethal means counseling is effective [8]. Thus, providing healthcare providers with specific training could improve culturally appropriate firearm screening efforts and lethal means counseling and reduce the number of firearm fatalities. Moreover, increasing the frequency of firearm screening and lethal means counseling among healthcare providers may help to normalize conversations on this topic.

### Limitations and Future Directions

This study was not without limitations. Participants were not asked to provide context on how and why they were screened within different settings. It is possible respondents were only screened for firearms at specific types of visits with their providers (e.g., well-child visits). Furthermore, data on firearm screening was retrospective and did not include a time period, so respondents were asked to recall past experiences with healthcare providers which may have resulted in response and recall biases. The sample size was limited, particularly in the AIAN sample, and therefore, findings should be interpreted with caution, and replication is needed. We recommend future studies examine the context surrounding firearm screening and expand upon studies from the healthcare provider perspective. Another limitation of the present study is that it did not examine how factors associated with structural racism may impact rates of screening. Future research should examine Black and AIAN’s experiences of racism within the healthcare setting to better understand how it may be related to low rates of firearm access screening. Additionally, the present study used a single item to assess firearm access, which may have resulted in underreporting. The study examined screening among the total sample and did not examine who may or may not have had the opportunity to be screened. Future research should seek to understand how access—or lack of access—to physical and mental healthcare may impact rates of screening. Lastly, the study did not examine attitudes on firearm screening and lethal means counseling. Future research should seek to better understand the attitudes and experiences of Black and AIAN individuals when it comes to firearm screening and lethal means counseling, to reduce barriers and promote adherence with recommendations.

In addition to the forementioned future directions, professional organizations play an important role in advancing and disseminating this work. Although groups like the AAP, the American College of Surgeons, and others have promoted routine screening for firearm access, they have been limited in their discussion of firearm screening among racial and ethnic minorities. Professional organizations should promote secure firearm storage practices among Black and AIAN individuals and advocate for funding to support trainings and access to secure storage devices for these communities.

## Conclusions

Findings from this study support previous research illustrating that healthcare providers rarely screen for firearm access and that individuals who identify as Black are being screened at disproportionately lower rates than White individuals, despite their high risk for firearm fatalities. An important first step is to identify and address the barriers among healthcare providers related to firearm access screening and lethal means counseling. Culturally congruent and streamlined training on firearm access screening and lethal means counseling for all healthcare professionals in hospitals, clinics, and community settings can potentially contribute to reducing the number of individuals injured or killed by a firearm. The findings of this study highlight major opportunities for healthcare providers to boost efforts to lower firearm-related injuries and deaths through universal firearm access screening and lethal means counseling, which in turn may help to normalize conversations around secure firearm storage and ultimately save lives.

## Data Availability

Data may be made available upon reasonable request to the author.

## Code Availability

Code may be made available upon reasonable request to the author.
